# A dose-volume histogram based decision-support system for dosimetric comparison of radiotherapy treatment plans

**DOI:** 10.1186/s13014-015-0569-3

**Published:** 2015-12-29

**Authors:** J. C. L. Alfonso, M. A. Herrero, L. Núñez

**Affiliations:** Center for Information Services and High Performance Computing (ZIH), Technische Universität Dresden, Nöthnitzer Str. 46, Dresden, 01062 Germany; Department of Applied Mathematics, Faculty of Mathematical Sciences, Universidad Complutense de Madrid (UCM), Ciudad Universitaria, Plaza Ciencias 3, Madrid, 28040 Spain; Radiophysics Department, Hospital Universitario Puerta de Hierro (HUPH), Calle Manuel de Falla 1 Majadahonda, Madrid, 28222 Spain

**Keywords:** Decision-support system, Plan evaluation, Radiation dose distribution index, Dose-volume histograms, Radiotherapy treatment planning

## Abstract

**Background:**

The choice of any radiotherapy treatment plan is usually made after the evaluation of a few preliminary isodose distributions obtained from different beam configurations. Despite considerable advances in planning techniques, such final decision remains a challenging task that would greatly benefit from efficient and reliable assessment tools.

**Methods:**

For any dosimetric plan considered, data on dose-volume histograms supplied by treatment planning systems are used to provide estimates on planning target coverage as well as on sparing of organs at risk and the remaining healthy tissue. These partial metrics are then combined into a dose distribution index (DDI), which provides a unified, easy-to-read score for each competing radiotherapy plan. To assess the performance of the proposed scoring system, DDI figures for fifty brain cancer patients were retrospectively evaluated. Patients were divided in three groups depending on tumor location and malignancy. For each patient, three tentative plans were designed and recorded during planning, one of which was eventually selected for treatment. We thus were able to compare the plans with better DDI scores and those actually delivered.

**Results:**

When planning target coverage and organs at risk sparing are considered as equally important, the tentative plan with the highest DDI score is shown to coincide with that actually delivered in 32 of the 50 patients considered. In 15 (respectively 3) of the remaining 18 cases, the plan with highest DDI value still coincides with that actually selected, provided that organs at risk sparing is given higher priority (respectively, lower priority) than target coverage.

**Conclusions:**

DDI provides a straightforward and non-subjective tool for dosimetric comparison of tentative radiotherapy plans. In particular, DDI readily quantifies differences among competing plans with similar-looking dose-volume histograms and can be easily implemented for any tumor type and localization, irrespective of the planning system and irradiation technique considered. Moreover, DDI permits to estimate the dosimetry impact of different priorities being assigned to sparing of organs at risk or to better target coverage.

## Background

A radiotherapy treatment plan is expected to uniformly irradiate a selected planning target volume (PTV), while at the same time minimizing radiation-induced damage to organs at risk (OARs) and the remaining volume at risk (RVR) [1–3]. To that end, a prescription dose (*D*_*p*_) to be delivered at the PTV should be defined, whereas radiation doses at OARs and RVRs should be kept as low as possible. To address that issue, computerized treatment planning systems (TPS) are used to simulate a number of tentative plans. For any such plan, the TPS provides dose-volume histograms (DVHs) for the PTV and each OAR delineated, as well as the isodose curves over the whole treatment domain. The choice of the treatment plan is then usually made, regardless of further physical and radiobiological considerations, mainly by inspection of the DVHs and isodose curves obtained for each tentative plan considered.

It is widely accepted that the decision-making process just recalled is highly subjective. Actually, considerable skills are required to pick up the most suitable tentative plan from a mere inspection of the DVHs and isodose curves of competing plans, since these may look quite similar to the naked eye. During the last decades, a number of approaches have been proposed to improve the choice of treatment plans. However, and in spite of the large efforts made so far, no widely accepted quantitative decision-support tool seems to be currently in clinical use [3, 4]. A standard approach towards deriving such decision-support tools has consisted in proposing physical-based quantitative metrics. Such metrics assign a score to a dosimetric plan according to its compliance with specified physical criteria [3]. Paramount among them, the conventionally used homogeneity index (HI) is defined as the ratio between the maximum radiation dose in the PTV and the prescription dose [5, 6]. In addition, alternative indices of dose homogeneity on the target have been proposed [7–10], which however fail often to accurately account for dose homogeneity within the PTV [7, 11]. On the other hand, a conformity index (CI) was first proposed in the Radiation Therapy Oncology Group (RTOG) radiosurgery guidelines [5], where it was defined as the ratio of the prescription isodose volume to the target volume. However, CI can yield false positives in extreme cases of nonconcordance between the target volume and isodose curves [6, 12]. Other conformity indices have been proposed [13–18], but practical limitations to their use have been pointed out in ICRU reports [1–3]. A major drawback of most of such indices is that they do not consider the overall dosimetric information provided by DVHs. As a matter of fact, a consensus has been built to consider DVHs as key indicators of the compliance with clinical requirements, and for that reason DVHs are routinely provided by the TPS.

The previous figures of merit provide a measure of target coverage and conformity only, without assessing organs at risk and healthy tissue sparing. This is a serious drawback, since normal tissue morbidity often represents the main limiting factor to radiation delivery and treatment outcomes [19, 20]. For that reason, several volume-based indices have been proposed to estimate such effects. These include, the healthy tissue overdosage factor (HTOF) [6], healthy tissue conformity index (HTCI) [6, 15] and critical organ-scoring index (COSI) [21]. Moreover, dose conformality at the PTV and OARs has also been simultaneously quantified [6, 22–24]. However, questions have been raised about the suitability of such indices to account for cold and hot spots (regions receiving particularly low or high radiation doses) in the PTV and OARs respectively [6, 7, 24].

It follows from our previous remarks that a single assessment index, accounting for a particular clinical aspect during planning, may not be suitable to compare tentative plans since it might ignore other key dosimetric issues. For this reason, indices have been proposed that provide combined scores for several treatment requirements. For instance, an overall quality factor (QF) and a unified dosimetry index (UDI) have been defined as weighted combinations of some of the indices mentioned before [25, 26]. However, the difficulty in interpreting the overall score thus obtained sets serious limitations to their practical use. Moreover, the figures of merit proposed often depend on many parameters [21, 24, 27], provide ambiguous scoring due to averaging effects [23, 25] and are not easy to implement in clinical routine [26, 28].

The indices recalled above are purely physical quantities based on dose-volume constraints. Radiobiological indices, such as tumor control probability (TCP) and normal tissue complication probability (NTCP), aim instead at estimating the effects of radiation on pathological and healthy tissues resulting from a given dose distribution. A large range of TCP [29–31] and NTCP [32–36] models have been reported, but recommendations in the ICRU reports suggest that they should be used with caution in clinical practice [1–3]. In particular, a major limitation to their use stems from the difficulty to estimate tissue-dependent parameter values to characterize the radiobiological response of tumors and organs involved. In many situations of clinical interest, such parameters have only been estimated in vitro or remain unknown.

## Methods

### Dose distribution index (DDI)

A dosimetric comparison system, the dose distribution index (DDI), is proposed to assist in the choice of radiotherapy treatment plans for any given cancer patient. DDI simultaneously takes into account key dosimetric issues as dose coverage, conformity and homogeneity over the PTV, as well as sparing of OARs and the RVR. Only standard information provided by the TPS, namely the DVHs and the prescription dose on the PTV, are required to grade and evaluate tentative plans. Moreover, one can readily estimate the separate impact on the PTV coverage and sparing of OARs and RVRs of any such modification, and thus use this information as a guidance to improve a tentative treatment plan.

**Fig. 1:**
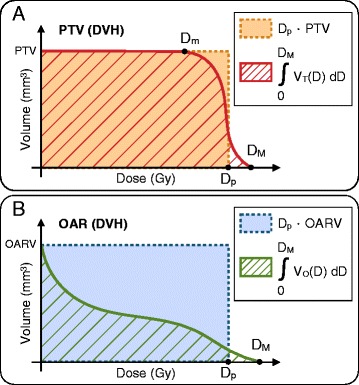
Schematic representation of dose-volume histograms. **a** A DVH corresponding to a PTV and **b** an OAR or the RVR. The factors involved in the formulation of *I*_*T*_, *I*_*O*_ and *I*_*R*_ are also indicated, see (1), (2) and (3) respectively

The proposed decision-support system is defined as a weighted sum of three components, which quantify the overall quality of a dosimetric plan. The first of those components assesses the dose coverage, conformity and homogeneity on the planning target, and it is defined as follows:

(1) $$ I_{T} = \left(1 - \left| 1- \left(\frac{\int\limits_{0}^{D_{M}} V_{T}(D) dD}{D_{p} \cdot PTV} \right) \cdot \left(\frac{D_{m}}{D_{M}} \right) \right| \right),  $$

where PTV is the planning target volume, *D*_*p*_ is the prescription dose on the PTV, *D*_*m*_ is the maximum dose received at least by 100 % of the PTV, *D*_*M*_ is the maximum dose in the PTV and *V*_*T*_(*D*) represents the DVH curve corresponding to the target. More precisely, this index computes the ratio between the areas under the DVH curve for any tentative plan considered and that corresponding to an ideal plan of constant *D*_*p*_ uniformly delivered on the PTV, see Fig. 1(a). The ratio (*D*_*m*_/*D*_*M*_) is included in (1) to account for dose homogeneity in the PTV. Alternatively, and depending on clinical requirements, this ratio might be taken as (*D*_95_/*D*_5_), where *D*_5_ and *D*_95_ are the dose received by the 5 and 95 % of the PTV respectively [7, 11]. Notice that as far as the PTV is considered, an ideal plan ensuring perfect dose coverage would yield a score *I*_*T*_=1.

We argue in a similar way to estimate the merit of a tentative treatment plan with respect to sparing of OARs and the RVR. In this case, a good plan would make close to zero the area under the DVH corresponding to such critical structures, see Fig. 1(b). The contributions to the DDI of OARs and RVR components are respectively formulated as follows:

(2) $$ I_{O} = \frac{1}{N} \sum\limits_{i=1}^{N} w_{Oi} \left(1- \left(\frac{\int\limits_{0}^{D_{Mi}} V_{Oi}(D) dD}{D_{p} \cdot OARV_{i}} \right) \cdot \left(\frac{D_{M}}{D_{p}} \right) \right)  $$

and

(3) $$ I_{R} = \left(1- \left(\frac{\int\limits_{0}^{D_{M}} V_{R}(D) dD}{D_{p} \cdot RVR} \right) \cdot \left(\frac{D_{M}}{D_{p}} \right) \right),  $$

where *D*_*M*_ is the maximum dose in each structure involved, *N* is the number of OARs considered, OARV is the volume of a given OAR, *V*_*O*_(*D*) and *V*_*R*_(*D*) represent the DVH curves of a given OAR and the RVR respectively. The ratio (*D*_*M*_/*D*_*p*_) is included to weight the deviation of the maximum dose received by OARs and the RVR from the prescription dose *D*_*p*_ on the PTV, the latter remaining constant for each competing plan considered for a same patient. The nonnegative weighting factors *w*_*Oi*_≤1.0 in (2) account for the relative clinical importance assigned to each OAR considered. In what concerns OARs and the RVR, an ideal plan would yield *I*_*O*_=*I*_*R*_=1, which would result in no irradiation at all on such critical volumes.

The DDI decision-support system is given by:

(4) $$ DDI = \frac{1}{3} \left(w_{T} I_{T} + w_{O} I_{O} + w_{R} I_{R} \right),  $$

where *w*_*T*_, *w*_*O*_ and *w*_*R*_ are nonnegative weighting parameters, each ranging between zero and one, that make precise the relative clinical priority assigned to the indices (1), (2) and (3) during the treatment planning process. These parameters allow to account for the relative importance assigned to target coverage and organs at risk sparing, from highly important (weight factor equal to 1) to less important (weight factor close to 0). In particular, if weights are selected equal 1 then all the structures involved are considered as equally important. In that manner, the DDI encodes the contributions from all three components into a unified score as defined by equation (4). Notice that an ideal plan would give *DDI*=1, which corresponds to uniform delivery of the *D*_*p*_ to the PTV and no irradiation at all in OARs and the RVR. The previous formulation can be easily extended to include several PTVs, similarly to what has been done for OARs in (2). In fact, this may be the case when a heterogeneous planning target is considered, and non-homogeneous radiation doses are prescribed [10, 28, 37]. For convenience of the reader, we summarize in Table 1 the notation used in the formulation of this decision-support system.

**Table 1:** Description of the notation used in the formulation of the DDI decision-support system

Symbol	Description
*D*_*p*_	Prescription radiation dose on the PTV
*D*_*M*_	Maximum dose in each structure delineated
*D*_*m*_	Maximum dose received at least by 100 % of the PTV
*N*	Number of OARs considered
PTV	Planning target volume
OARV _*i*_	Volume of the ith OAR considered
RVR	Remaining volume at risk
*V*_*T*_(*D*)	DVH curve of the PTV
*V*_*Oi*_(*D*)	DVH curve of the ith OAR considered
*V*_*R*_(*D*)	DVH curve of the RVR
*w*_*T*_	Weight associated to *I*_*T*_
*w*_*O*_	Weight associated to *I*_*O*_
*w*_*Oi*_	Weight associated to the ith OAR considered
*w*_*R*_	Weight associated to *I*_*R*_

To illustrate how the DDI would work in clinical practice, we have performed a retrospective study over fifty brain cancer patients already treated at Hospital Universitario Puerta de Hierro in Madrid (HUPH). For each patient, three tentative plans were designed with the same TPS during the planning process. Out of the three competing plans considered, one was the selected by clinicians and delivered to the patient. We have then compared in each case the applied treatment plan with the tentative plan achieving the highest DDI score.

### Patients’ characteristics

Fifty brain cancer patients randomly selected and previously treated by external beam radiotherapy at HUPH between the years 2012 and 2014 were considered for the present study. In particular, patients with brain tumors were selected due the large amount of OARs involved, and the consequent difficulty of global evaluation of PTV coverage and OARs sparing. The patients were divided in three different groups, based on tumor location and malignancy, as follows: (i) 25 patients with centrally located benign tumors (13 Meningiomas, 6 Pituitary Adenomas, 5 Acoustic Neuromas and 1 Facial Nerve Neuroma) representing 50 % of the sample; (ii) 10 patients with centrally located malignant tumors (4 Malignant Craniopharyngiomas, 2 Cavum Carcinomas, 3 Chondrosarcomas and 1 Clear Cell Carcinoma (CCC) of the Base of the Tongue) making up for 20 % of the total, and (iii) 15 patients with peripheral benign and malignant tumors (12 Brain Metastasis, 2 Astrocytomas and 1 Meningioma) which correspond to 30 % of the cases considered.

### Volume definitions

PTVs, OARs and RVRs were defined as recommended by ICRU reports [1–3]. The gross tumour volume (GTV) was delineated on all relevant slices of a planning CT scan. The margins were then expanded isotropically to allow for microscopic invasion, thus giving raise to the clinical target volume (CTV). This last is further enlarged to allow for organ motion and set-up error. The resulting structure of interest is then defined as the planning target volume (PTV). The organs selected as OARs including the Brainstem, Eyes, Crystalline Lens, Lacrimal Glands, Retinas, Optic Chiasm, Hypothalamus, Cochleas, Spinal Cord, Parotid Glands, Pituitary Gland, Optic Nerves, Optic Tracts and Cranial Nerves (Trigeminal, Facial and Vestibulocochlear Nerves) were contoured on the relevant transverse CT slices of the planning system.

### Treatment planning

For each patient, three different tentative plans using three-dimensional conformal radiation therapy (3D-CRT) or sliding windows intensity-modulated radiation therapy (IMRT) were simulated. The dose-volume planning prescription on the target required that at least 95 % of the PTV had to receive no less than 95 % of the prescription dose. This last was established at 50.4 Gy in 28 daily fractions (5 days a week) of 1.8 Gy for conventional fractionation protocols (in twenty-nine out of fifty patients, 58 % of total), and between 12 Gy–18 Gy in a single session for stereotactic radiosurgery (in the remaining twenty-one patients, 42 % of total). The dose planification system was an iPLAN RT Dose 4.1.1 (BrainLAB AG, Germany). Sets of three alternative treatment plans, with the same radiotherapy technique, structures of interest and clinical requirements, were prepared for each patient. Out of the three tentative plans considered, one was selected and then delivered to the patient. For all tentative plans the DVHs corresponding to each volume delineated were extracted from the treatment planning system for computation of the DDI.

## Results

**Fig. 2:**
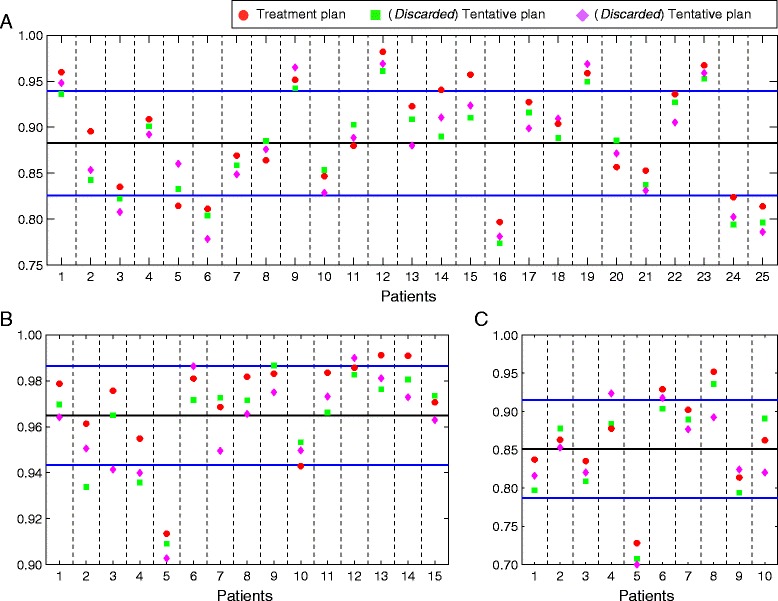
Plots of the DDI scores for each set of three tentative plans corresponding to the fifty brain cancer patients considered. Applied treatment plan in red, and discarded tentative plans in green and purple. **a** 25 centrally located benign tumors. **b** 15 peripherally located benign and malignant tumors. **c** 10 centrally located malignant tumors. Mean DDI scores (black solid lines) and standard deviations (blue solid lines) are also represented

In Table 2 the resulting mean *I*_*T*_, *I*_*O*_, *I*_*R*_ and DDI scores, as well as standard deviations for the overall set of tentative plans are provided. Weighting factors in components (2) and (4) were set equal to one, so that the PTV coverage and sparing of OARs and RVRs are considered as equally important. The lowest and highest DDI scores, corresponding to the minimum and maximum deviations from an ideal dosimetric plan, were 0.70 and 0.98 respectively. A comparison of mean *I*_*T*_, *I*_*O*_, *I*_*R*_ and DDI scores, as well as standard deviations for all tentative plans corresponding to patients with centrally and peripherally located tumors is also shown in Table 2. From the results displayed there, we observe that tentative plans for peripheral targets are in average closer to an ideal dosimetric plan than those for centrally located targets with respect to PTV coverage and sparing of OARs and RVRs. Figure 2 shows the DDI scores for all tentative plans for each group of patients considered, as well as the corresponding means and standard deviations. The small differences observed on the DDI values between competing plans in some patients are nearly imperceptible to the naked eye and thus difficult to detect and quantify. However, the DDI was able to significantly discriminate between similar-looking DVHs when scoring the corresponding tentative plans.

**Table 2:** Scoring results corresponding to all tentative plans for the fifty brain cancer patients considered and differences between centrally and peripherally located tumors

	*I*_*T*_		*I*_*O*_		*I*_*R*_		DDI	
	Mean	Std	Mean	Std	Mean	Std	Mean	Std
T	0.87	0.12	0.85	0.11	0.93	0.04	0.88	0.09
CL	0.86	0.11	0.83	0.10	0.94	0.03	0.87	0.08
PL	0.93	0.07	0.97	0.02	0.96	0.02	0.95	0.04

Mean *I*_*T*_, *I*_*O*_, *I*_*R*_ and DDI scores, as well as standard deviations are represented for all tentative plans (T) and centrally and peripherally located tumors (labelled as CL and PL respectively)

Concerning the degree of coincidence between the applied treatment plan (ATP) and the tentative plan (DDIP) achieving the highest DDI score, we separately analyzed each group of patients classified by their tumor location and malignancy. In the cases of (i) centrally located benign tumors, (ii) centrally located malignant tumors and (iii) peripherally located benign and malignant tumors, we obtained that ATP and DDIP agree in 17 out of the 25, 6 out of the 10, and 9 out of the 15 patients respectively, see Fig. 2. Altogether, this results in an agreement between ATP and DDIP for 32 out of the 50 patients in this study under the assumption that PTV coverage and sparing of OARs and the RVR are considered as equally important.

**Fig. 3:**
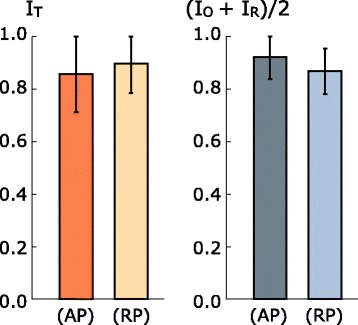
Comparison of scoring results for the cases where ATP and DDIP did not coincide. The mean *I*_*T*_ and joint *I*_*O*_ and *I*_*R*_ defined as (*I*_*O*_+*I*_*R*_)/2 scores and standard deviations are provided for the ATPs (label AP) and remaining tentative plans (label RP)

To shed some light on the clinical choices made for the patients where ATP and DDIP did not coincide, we separately analyzed the DDI components for the planning target and critical structures in such cases. Figure 3 shows a comparison of the mean *I*_*T*_ and $\left (I_{O} + I_{R}\right)/2$ scores, which respectively estimate the quality of PTV coverage and critical structures sparing, for ATPs and the remaining competing plans for patients where ATP and DDIP did not coincide. We observe that, although the APTs were not in average the choices providing better PTV coverage, they yielded lower irradiation of critical structures. More precisely, in 15 out of the 18 cases of no coincidence $\left (I_{O} + I_{R}\right)/2$ was higher in the APTs compared to other competing plans, while *I*_*T*_ was higher in the ATPs of the remaining 3 patients.

**Fig. 4:**
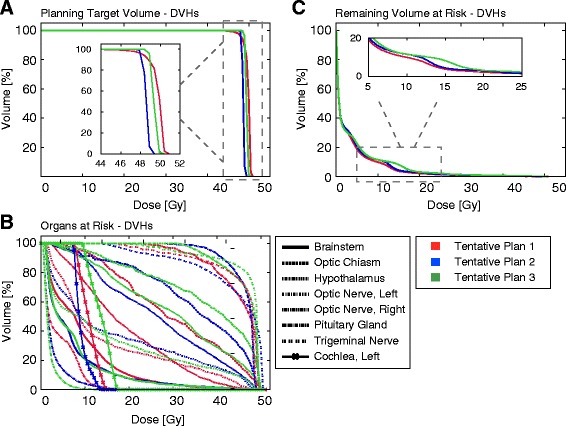
DVHs corresponding to the PTV (**a**), OARs (**b**) and RVR (**c**) for the three tentative plans of a patient diagnosed with a centrally located Meningioma. The *D*_*p*_ on the PTV was set equal to 50.4 Gy in 28 daily fractions (5 days per week) of 1.8 Gy

To gain further insight in one of the discrepancy cases, we selected a patient diagnosed with a centrally located Meningioma, where the ATP and DDIP did not coincide. Figure 4 shows the DVHs for the PTV and nearby OARs corresponding to the three different tentative plans prepared for that patient. OARs receiving low radiation doses as Eyes, Lacrimal Glands, Crystalline Lens and Retinas have not been represented for visualization simplicity, but all of them were considered in the calculation of the DDI scores. A quick glance at Fig. 4 reveals that in all tentative plans the target coverage is significantly high. However, what is unclear is which plan best provides target coverage and OARs sparing. In fact, when DVHs for all competing plans are superposed as shown in Fig. 4, the high number of OARs involved makes difficult to select the most suitable plan by mere inspection.

In Table 3 the *I*_*T*_, *I*_*O*_, *I*_*R*_ and DDI scores for such tentative plans are provided. We see that if PTV coverage is given maximum priority, the best choice corresponds to Plan 3, followed by Plans 2 and respectively 1. On the contrary, when lowering radiation doses on OARs and the RVR are selected as the primary clinical goal, the most suitable choice would be Plan 1, followed by Plans 2 and 3 respectively. Thus, consideration of partial DDI scores reveals that increasing (respectively decreasing) the target coverage was detrimental (respectively favorable) to sparing of OARs and the RVR. Although based on the DDI scores the Plan 2 provided the best balance between target coverage and sparing of critical structures, the Plan 1 was actually selected for treatment. This is consistent with highest priority having been assigned to sparing of OARs and the RVR at that time. For instance, a choice of relative weighting factors in (4) as *w*_*T*_=0.5, *w*_*O*_=1.0 and *w*_*R*_=1.0 would have suggested that choice.

**Table 3:** Scoring results for a patient diagnosed with a centrally located Meningioma

	*I*_*T*_	*I*_*O*_	*I*_*R*_	DDI
Plan 1	0.832	0.821	0.948	0.867
Plan 2	0.923	0.789	0.934	0.882
Plan 3	0.934	0.776	0.918	0.876

*I*_*T*_, *I*_*O*_, *I*_*R*_ and DDI scores for the set of three tentative plans simulated. The table shows that Plan 1 (ATP) provides the highest sparing of OARs and RVR, while Plan 3 results in the best PTV coverage. However, the Plan 2 has the highest DDI score

## Discussion

The availability of commercial TPS allows radiophysicists and radiation oncologists to virtually explore different tentative plans, and to perform successive modifications of a starting plan, for each radiotherapy patient. However, to this day the choice of treatment plans is mostly made by side-by-side comparison of DVHs and isodose curves for each tentative plan considered. Since differences between them are usually small, any selection made by inspection, as it is generally the case, may not be easy to justify on clinical terms. Therefore, the development of clinically based quantitative scoring indices could be quite useful as decision-support tools to assist in making clinical choices. For that purpose, several indices for scoring dosimetric plans have been proposed and shortly recalled at the ‘Background’ Section.

Bearing these facts in mind, we have introduced here a decision-support system, termed dose distribution index (DDI), to compare different tentative plans and to estimate the consequences of modifications on any treatment plan under consideration. The DDI makes use of the overall dosimetric information provided by the DVHs to simultaneously evaluate the target coverage and sparing of critical structures. Moreover, this evaluation tool can be easily extended to include several PTVs, as well as multiple dose coverage objectives on heterogeneous tumors. In our view, such features represent a comparative advantage with respect to previous combined indices. For instance, the unified dosimetry index (UDI) [25] makes use of four dosimetric objectives of dose coverage, conformity, homogeneity, and dose gradient on the PTV. Although the UDI provides a useful tool for ranking tentative plans, this figure of merit relies in specific dose-volume constraints instead to consider the overall dosimetric information provided by the DVHs. Moreover, UDI does not directly account for sparing of critical structures and does not seem easy to use when multiple target dose coverage objectives are specified. To consider a further example, a histogram analysis in radiation therapy (HART) computational tool has been proposed in [26]. This software incorporates various physically and biologically based indices for plan evaluation. Whereas HART is a complete and suitable program to compare tentative plans, the results are prone to the intrinsic limitations of the indices therein considered, as reported in the ICRU reports [1–3].

We point out that the DDI is very sensitive to small dosimetric variations between tentative plans and distinctly discriminates situations where DVHs look quite similar to the naked eye. Furthermore, it may be also used to compare tentative plans obtained from any TPS irrespective of tumor type and location, and can be formulated for any irradiation technique that provides DVHs. At the practical level, computing the DDI is straightforward, and that decision-support system can be easily displayed on TPS simultaneously to the visualization of the corresponding DVHs. In addition, it may readily provide the separate values *I*_*T*_, *I*_*O*_ and *I*_*R*_, as well as those for each single OAR, which could be a useful complement to the DVH analysis.

Although the DDI can be used to globally assess the quality of any treatment plan, its main practical interest could be to compare tentative plans for a same patient. In fact, depending on diverse factors as tumor malignancy and location, curative or palliative purposes or overall patient’s medical history, a dosimetric plan can have a lower DDI score compared to other treatment plans for different patients, and yet be the best possible choice that satisfies concrete clinical requirements and treatment goals in that particular case. On the other hand, for a given patient different planners may well pursue different, and occasionally conflicting, goals. For instance, dose coverage and conformity on the PTV may be given highest priority in some cases, while palliative considerations may require of minimal damage to OARs and the RVR in others. DDI is not designed to change such priorities, but rather to help clinicians to precisely assess the consequences of their choices during treatment planning.

To give an idea of what DDI can accomplish, we have presented a retrospective study, corresponding to fifty patients treated at HUPH of centrally and peripherally located benign and malignant brain tumors. In each case, three tentative plans were simulated and recorded, out of which one was eventually delivered to the patient. We then compared the applied treatment plan (ATP) with that which provides the highest DDI score (DDIP). We observed that, under the assumption that PTV covering and OARs and RVRs sparing are equally important, ATP and DDIP coincided in 32 of the 50 cases. Discrepancies between ATP and DDIP in the remaining 18 cases may well result from different clinical priorities being attributed to critical structures during planning. In fact, in those cases ATP and DDIP also coincide, provided that different orders of priorities (for instance, deeming OARs sparing as the most important goal) are established instead.

We finally remark on the modular character of the quantitative index provided. In particular, DDI can be combined with multi-parameter optimization algorithms and included in objective functions during radiotherapy planning processes. In fact, considerable attention is being paid to the design of optimization methods to obtain dose distributions satisfying specific criteria as high tumor coverage and low radiation dose deposition on OARs [3, 38–40]. However, no method has been found as yet to computationally find a plan that satisfies current clinical and logistical requirements in an optimal way. We expect that implementing the DDI decision-support system in current TPS would improve reliability standards, by precisely quantifying the impact of planner subjectivity in assessing and selecting radiotherapy treatment plans.

## Conclusions

In this work a decision-support system has been developed to compare and evaluate different tentative plans during radiotherapy treatment planning. The proposed DDI is a straightforward and non-subjective system to assess and compare competing tentative plans. The DDI takes into account the overall dosimetric information provided by DVHs and not only that given by selected dose-volume constraints. Moreover, DDI can be easily computed irrespective of the TPS in clinical use, tumor type and localization, and irradiation technique. This decision-support tool is not meant to be a replacement for planners’ clinical decisions. Instead, it is intended to provide clinical personnel with a precise quantitative assessment of the impact of dosimetric changes over any starting tentative plan that they might consider.

## Abbreviations

3D-CRT: three-dimensional conformal radiation therapy
ATP: applied treatment plan
CCC: clear cell carcinoma
CI: conformity index
COSI: critical organ scoring index
CTV: clinical target volume
DDI: dose distribution index
DDIP: tentative plan achieving the highest DDI score
DVH: dose-volume histogram
GTV: gross tumour volume
CT: computed tomography
GUI: graphical user interface
HI: homogeneity index
HTCI: healthy tissue conformity index
HTOF: healthy tissue overdosage factor
HUPH: Hospital Universitario Puerta de Hierro, Madrid
ICRU: International Commission on Radiation Units and Measurements
IMRT: intensity-modulated radiation therapy
NTCP: normal tissue complication probability
PTV: planning target volume
OAR: organ at risk
QF: quality factor
RTOG: Radiation Therapy Oncology Group
RVR: remaining volume at risk
TPS: treatment planning system
TCP: tumor control probability
UDI: unified dosimetry index
